# Real and Pseudo Pedestrian Detection Method with CA-YOLOv5s Based on Stereo Image Fusion

**DOI:** 10.3390/e24081091

**Published:** 2022-08-08

**Authors:** Xiaowei Song, Gaoyang Li, Lei Yang, Luxiao Zhu, Chunping Hou, Zixiang Xiong

**Affiliations:** 1 School of Electronic and Information, Zhongyuan University of Technology, Zhengzhou 450007, China; 2 Dongjing Avenue Campus, Kaifeng University, Kaifeng 475004, China; 3 School of Electrical and Information Engineering, Tianjin University, Tianjin 300072, China; 4 Department of Electrical and Computer Engineering, Texas A&M University, College Station, TX 77843, USA

**Keywords:** stereo image fusion, pedestrian detection, CA, YOLOv5s, pseudo pedestrian

## Abstract

With the development of convolutional neural networks, the effect of pedestrian detection has been greatly improved by deep learning models. However, the presence of pseudo pedestrians will lead to accuracy reduction in pedestrian detection. To solve the problem that the existing pedestrian detection algorithms cannot distinguish pseudo pedestrians from real pedestrians, a real and pseudo pedestrian detection method with CA-YOLOv5s based on stereo image fusion is proposed in this paper. Firstly, the two-view images of the pedestrian are captured by a binocular stereo camera. Then, a proposed CA-YOLOv5s pedestrian detection algorithm is used for the left-view and right-view images, respectively, to detect the respective pedestrian regions. Afterwards, the detected left-view and right-view pedestrian regions are matched to obtain the feature point set, and the 3D spatial coordinates of the feature point set are calculated with Zhengyou Zhang’s calibration method. Finally, the RANSAC plane-fitting algorithm is adopted to extract the 3D features of the feature point set, and the real and pseudo pedestrian detection is achieved by the trained SVM. The proposed real and pseudo pedestrian detection method with CA-YOLOv5s based on stereo image fusion effectively solves the pseudo pedestrian detection problem and efficiently improves the accuracy. Experimental results also show that for the dataset with real and pseudo pedestrians, the proposed method significantly outperforms other existing pedestrian detection algorithms in terms of accuracy and precision.

## 1. Introduction

Pedestrian detection is an important branch of object detection, having received wide attention in the past two decades [1]. The purpose of pedestrian detection is to find all possible pedestrians in the input image and output the location of pedestrian in the image. Pedestrian detection can be widely used in areas such as safety monitoring and automatic driving, where the accuracy of pedestrian detection is crucial [2].

Pedestrian detection technology has developed from traditional hand-assisted feature detection [3,4,5] to modern deep learning-based feature detection [6,7,8,9]. Traditional pedestrian detection algorithms require the manual design of filters and features, such as Gabor filter, gradient-based feature, channel feature, etc., according to statistical or prior knowledge of the designer. Cheng et al. proposed a pedestrian detection method using a sparse Gabor filter which is designed according to the learned texture features from some manually selected typical images of pedestrian [10]. Dalal proposed a pedestrian detection method using edge features extracted by a histogram of oriented gradient (HOG), which is obtained by the calculation and statistics of HOG in some manually selected local image areas [5]. Dollar et al. proposed a pedestrian detection method using channel features extracted by the integral of some manually selected registered image channels [11]. These traditional pedestrian detection algorithms are time consuming and laborious due to the manual intervention, with relatively low detection accuracy and efficiency.

With the development of convolutional neural networks, the effect of pedestrian detection has been pushed to an unprecedentedly high level by the modern deep learning-based pedestrian detection algorithms [12,13]. Modern pedestrian detection algorithms based on deep learning can autonomously learn and extract features of pedestrian, with high detection accuracy and efficiency. Many challenging problems have been well solved [14]. For instance, Zhang et al. solved the problem of small-scale pedestrian detection with asymmetric multi-stage CNNs [15]. Xu et al. solved the efficiency problem of pedestrian detection through the model reconstruction and pruning of YOLOv3 network [16]. Lin et al. solved the robustness problem of obscured pedestrian detection with multi-grained deep feature learning [17]. Li et al. solved the effectiveness problem of pedestrian detection in hazy weather with a weighted combination layer, which combines multi-scale feature maps with a squeeze and excitation block [18]. However, the elimination problem of false positive samples in pedestrian detection has not been solved yet.

The false positive samples include trash cans, traffic lights, trees and people printed on flat surfaces. Since these false positive samples have similar characteristics to pedestrians, they are always incorrectly detected as pedestrian by most pedestrian detection algorithms [19]. The incorrect detection of false positive samples, such as trash cans, traffic lights and trees has been solved through network improvement [20,21,22]. However, the incorrect detection of people printed on flat surfaces has not been well solved because printed people have almost exactly the same characteristics as pedestrians. There are mainly two types of pedestrians printed on flat surfaces: pseudo pedestrian in a 2D plane with background (PPWB) and pseudo pedestrian in a 2D plane with no background (PPWNB), which are collectively called pseudo pedestrians in this paper.

There is almost no difference between real and pseudo pedestrians in 2D features, so it is necessary to take advantage of 3D features to distinguish them. There have been some attempts to detect pedestrians with 3D information. Shakeri et al. collected 3D information contained in the left-view and right-view images by a binocular stereo camera, enhanced the image quality of the pedestrian area of interest by 3D information fusion, and thus improved the accuracy of pedestrian detection [23]. However, only 2D information is used in pedestrian detection, which cannot realize real and pseudo pedestrian detection. Wei et al. also captured 3D information included in the left-view and right-view images by a binocular stereo camera, took advantage of the complementary information of the left-view and right-view images, and solved the problem of obscured pedestrian detection [24]. Nevertheless, similar to Ref. [23], only 2D information is used in pedestrian detection, which cannot complete real and pseudo pedestrian detection as well. Zhao et al. acquired 3D information contained in the 2D image and depth map by a light field camera, and performed pedestrian detection according to the 3D information, including 2D information and depth information [25]. PPWB at the same depth as the background can be distinguished from the real pedestrian, while PPWNB not at the same depth as the background can still not be distinguished from the real pedestrian. Therefore, it is necessary to further solve the problem of pedestrian detection involving both PPWB and PPWNB.

In this paper, a real and pseudo pedestrian detection method with CA-YOLOv5s based on stereo image fusion is proposed. The proposed method is designed according to the constructed real and pseudo pedestrian detection bionic model based on human stereo vision. A binocular stereo camera is adopted to capture the left-view and right-view images of the pedestrian. The two-view images are respectively detected by the improved CA-YOLOv5s pedestrian detection algorithm to obtain the respective pedestrian regions. The detected pedestrian regions are stereo matched to obtain a feature point set, and the 3D spatial coordinates of the feature point set are calculated with Zhengyou Zhang’s calibration method. The mismatched feature points are eliminated, and a matched feature point set is reserved. The 3D features of the matched feature point set are extracted by random sample consensus (RANSAC) plane fitting, and the real and pseudo pedestrian detection is completed by the trained support vector machine (SVM) model. The proposed method can effectively solve the problem of pseudo pedestrian detection, and increase the accuracy as well.

The rest of the paper is organized as follow. In Section 2, we review some related works on the principle of human stereo vision and attention mechanism. In Section 3, we construct a real and pseudo pedestrian detection bionic model based on human stereo vision and propose a real and pseudo pedestrian detection method with CA-YOLOv5s based on stereo image fusion. In Section 4, we report the experimental results. In Section 5, we make a conclusion.

## 2. Related Works

### 2.1. Principle of Human Stereo Vision

**Figure 1:**
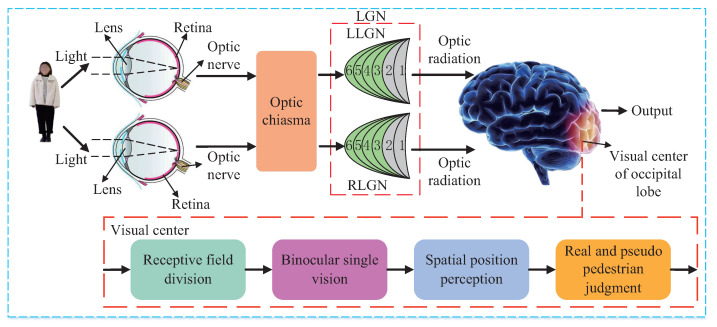
Pedestrian detection principle diagram of human stereo vision.

Human stereo vision can perfectly realize the real and pseudo pedestrian detection, so it is the biological theoretical basis of the proposed method in this paper. In human stereo vision system, as shown in Figure 1, the 3D pedestrian is imaged on the retina through human optical components such as lens, and the photoreceptor cells on the retina convert optical signals into bioelectrical signals which are transmitted to the optic chiasma through the optic nerve. The optic chiasma rearranges the signals and transmits them to the lateral geniculate nucleus (LGN), and the processed signals are sent to the visual center of the occipital lobe through optic radiation. In the visual center of the occipital lobe, the region of interest is extracted by the receptive field division, the binocular single vision is formed through fusion, the stereo vision is achieved through spatial position perception, and the real and pseudo pedestrian judgment is made accordingly.

When viewing an object, the optic chiasma rearranges the signals from the right visual field of the left eye and the right visual field of the right eye and transmits them to the left LGN (LLGN), and rearranges the signals from the left visual field of the left eye and the left visual field of the right eye and transmits them to the right LGN (RLGN) [26]. For LLGN, the light intensity $I_{L}^{r}$ of the optical signal perceived at the right visual field of the left retina $\left(x_{L}^{r},y_{L}^{r}\right)$ from the right visual field of the left eye at time *t* can be expressed by Equation (1), while the light intensity $I_{R}^{r}$ of the optical signal perceived at the right visual field of the right retina $\left(x_{R}^{r},y_{R}^{r}\right)$ from the right visual field of the right eye at time t can be expressed by Equation (2). For RLGN, the light intensity $I_{L}^{l}$ of the optical signal perceived at the left visual field of the left retina $\left(x_{L}^{l},y_{L}^{l}\right)$ from the left visual field of the left eye at time t can be expressed by Equation (3), while the light intensity $I_{R}^{l}$ of the optical signal perceived at the left visual field of the right retina $\left(x_{R}^{l},y_{R}^{l}\right)$ from the left visual field of the right eye at time t can be expressed by Equation (4).

(1) $$I_{L}^{r}\left(x_{L}^{r},y_{L}^{r},t\right)=k_{L}\int_{\lambda_{l}}^{\lambda_{h}}P_{L}\left(x_{L}^{r},y_{L}^{r},\lambda ,t\right)V_{L}\left(\lambda \right)d\lambda$$

(2) $$I_{R}^{r}\left(x_{R}^{r},y_{R}^{r},t\right)=k_{R}\int_{\lambda_{l}}^{\lambda_{h}}P_{R}\left(x_{R}^{r},y_{R}^{r},\lambda ,t\right)V_{R}\left(\lambda \right)d\lambda$$

(3) $$I_{L}^{l}\left(x_{L}^{l},y_{L}^{l},t\right)=k_{L}\int_{\lambda_{l}}^{\lambda_{h}}P_{L}\left(x_{L}^{l},y_{L}^{l},\lambda ,t\right)V_{L}\left(\lambda \right)d\lambda$$

(4) $$I_{R}^{l}\left(x_{R}^{l},y_{R}^{l},t\right)=k_{R}\int_{\lambda_{l}}^{\lambda_{h}}P_{R}\left(x_{R}^{l},y_{R}^{l},\lambda ,t\right)V_{R}\left(\lambda \right)d\lambda$$

Wherein $\left(x_{L}^{r},y_{L}^{r}\right)$ and $\left(x_{R}^{r},y_{R}^{r}\right)$ are the coordinates of the corresponding imaging points in the right visual field of the left and right retina, respectively; $\left(x_{L}^{l},y_{L}^{l}\right)$ and $\left(x_{R}^{l},y_{R}^{l}\right)$ are the coordinates of the corresponding imaging points in the left visual field of the left and right retina respectively; $k_{l}$ and $k_{r}$ are the adjustable coefficients of the left and right eye respectively; $P_{L}\left(x_{L}^{r},y_{L,}^{r}\lambda ,t\right)$ and $P_{R}\left(x_{R}^{r},y_{R}^{r},\lambda ,t\right)$ are the radiation power of light with wavelength λ received at $\left(x_{L}^{r},y_{L}^{r}\right)$ and $\left(x_{R}^{r},y_{R}^{r}\right)$ respectively; $P_{L}\left(x_{L}^{l},y_{L}^{l},\lambda ,t\right)$ and $P_{R}\left(x_{R}^{l},y_{R}^{l},\lambda ,t\right)$ are the radiation power of light with wavelength λ received at $\left(x_{L}^{l},y_{L}^{l}\right)$ and $\left(x_{R}^{l},y_{R}^{l}\right)$, respectively; $V_{L}\left(\lambda \right)$ and $V_{R}\left(\lambda \right)$ are the spectral response functions of the left and right eye, respectively; $\lambda_{h}$ and $\lambda_{l}$ are the upper and lower wavelength limits of human eye perception.

The optical signal causes ion exchange in the Na^+^-K^+^ ion pumps in the photoreceptor cells of the retina, resulting in a change in the electric potential, which is voltage [27]. Thus, the optical signals $I_{L}^{r}$ and $I_{R}^{r}$ at the right visual field of the left and right retina are converted into the bioelectrical signals $U_{L}^{r}$ and $U_{R}^{r}$ in the right visual field by photoelectric conversion (PEC), as expressed by Equations (5) and (6). The optical signals $I_{L}^{l}$ and $I_{R}^{l}$ at the right left field of the left and right retina are converted into the bioelectrical signals $U_{L}^{l}$ and $U_{R}^{l}$ in the left visual field by PEC, as expressed by Equations (7) and (8).

(5) $$U_{L}^{r}\left(x_{L}^{r},y_{L}^{r},t\right)=PEC\left(I_{L}^{r}\left(x_{L}^{r},y_{L}^{r},t\right)\right)$$

(6) $$U_{R}^{r}\left(x_{R}^{r},y_{R}^{r},t\right)=PEC\left(I_{R}^{r}\left(x_{R}^{r},y_{R}^{r},t\right)\right)$$

(7) $$U_{L}^{l}\left(x_{L}^{l},y_{L}^{l},t\right)=PEC\left(I_{L}^{l}\left(x_{L}^{l},y_{L}^{l},t\right)\right)$$

(8) $$U_{R}^{l}\left(x_{R}^{l},y_{R}^{l},t\right)=PEC\left(I_{R}^{l}\left(x_{R}^{l},y_{R}^{l},t\right)\right)$$

The bioelectrical signals $U_{L}^{r}$ and $U_{R}^{r}$ in the right visual field are transmitted to the optic chiasma (OC) through the optic nerve, where they are rearranged and sent to the LLGN. The bioelectrical signals received by the LLGN can be expressed by Equation (9). The bioelectrical signals $U_{L}^{l}$ and $U_{R}^{l}$ in the left visual field are transmitted to the optic chiasma through the optic nerve, where they are rearranged and sent to the RLGN. The bioelectrical signals received by the RLGN can be expressed by Equation (10).

(9) $$U_{\mathrm{LLGN}}\left(x^{r},y^{r},t\right)=OC\left(U_{L}^{r}\left(x_{L}^{r},y_{L}^{r},t\right)\cup U_{R}^{r}\left(x_{R}^{r},y_{R}^{r},t\right)\right)$$

(10) $$U_{\mathrm{RLGN}\;}\left(x^{l},y^{l},t\right)=OC\left(U_{L}^{l}\left(x_{L}^{l},y_{L}^{l},t\right)\cup U_{R}^{l}\left(x_{R}^{l},y_{R}^{l},t\right)\right)$$

The bioelectrical signals $U_{\mathrm{LLGN}}$ in the LLGN are sent to the left brain through optic radiation (OR). The bioelectrical signals $U_{LB}$ received by the left brain can be expressed by Equation (11), which represents the right visual field. The bioelectrical signals $U_{\mathrm{RLGN}}$ in the RLGN are sent to the right brain through optic radiation. The bioelectrical signals $U_{RB}$ received by the right brain can be expressed by Equation (12), which represent the left visual field.

(11) $$U_{LB}\left(x^{r},y^{r},t\right)=OR\left(U_{\mathrm{LLGN}}\left(x^{r},y^{r},t\right)\right)$$

(12) $$U_{RB}\left(x^{l},y^{l},t\right)=OR\left(U_{\mathrm{RLGN}}\left(x^{l},y^{l},t\right)\right)$$

In the visual center of the occipital lobe, the bioelectrical signals $U_{LB}$ and $U_{RB}$ are combined into a bioelectrical signal $U_{B}$ representing the whole visual field, which can be expressed by Equation (13).

(13) $$U_{B}\left(x,y,t\right)=U_{RB}\left(x^{l},y^{l},t\right)\cup U_{LB}\left(x^{r},y^{r},t\right)$$

Visual cortex cells only respond significantly to the bioelectrical signal $U_{B_{-}RF}$ in their receptive field (RF), as expressed by Equation (14).

(14) $$U_{B_{-}RF}\left(x_{RF},y_{RF},t\right)=RF\left(U_{B}\left(x,y,t\right)\right)$$

The bioelectrical signal $U_{B_{-}RF}$ has a hierarchical structure, in which different layers correspond to the different bioelectrical signals from the left and right eyes, respectively. The visual cortex of the brain fuses the layered bioelectrical signals $U_{B_{-}RF}$ in the receptive field to form a single object image, that is, binocular single vision, then the spatial position perception is realized, as expressed by Equation (15).

(15) $$I_{P}\left(X,Y,Z,t\right)=F\left(U_{B_{-}RF}\left(x_{RF},y_{RF},t\right)\right)$$

Finally, the real and pseudo pedestrian judgment is made by the brain according to the perceived stereo vision information $I_{P}$ and the judgment result is output, as expressed by Equation (16).

(16) $$\;\mathrm{Output}\;=J\left(I_{P}\left(X,Y,Z,t\right)\right)$$

With the above process, the real and pseudo pedestrian judgment is completed by the human stereo vision system.

### 2.2. Attention Mechanism

In the pedestrian detection network, more weight can be allocated to the pedestrian area and less weight to the background area through the focusing effect of the attention mechanism, so as to improve the accuracy of pedestrian detection and reduce the network model parameters.

According to its processing mechanism, the attention module can be divided into three types: spatial attention module, channel attention module and mixed attention module [28,29,30,31,32]. The spatial attention module carries out average pooling and maximum pooling in the channel direction at the same time using the spatial weight matrix. The spatial attention matrix is obtained by convolution, and a 2D spatial attention map is generated by the activation function, thus the spatial position that needs to be focused on is determined. Moreover, the attention mechanism has also been used in multimodal image fusion [33,34,35] to enhance the pedestrian detection, and has achieved promising results.

Typical channel attention module includes squeeze-and-excitation (SE) and efficient channel attention (ECA). SE samples the input image by global average pooling, learns the dependence to each channel by the shared multilayer perceptron (MLP), and generates the channel attention map by the activation function [28]. ECA improves the shared MLP part of SE, focusing on the interaction of each channel and its k neighborhood channels, and greatly reduces the network parameters [29]. The mixed attention module combines different kinds of attention. The convolutional block attention module (CBAM) and coordinate attention (CA) are the typical representatives. CBAM connects the channel attention module with the spatial attention module through convolution, and can obtain the spatial attention and channel attention joint optimized features [30]. CA embeds the location information into the channel attention module, and decomposes the channel attention module into two 1D feature coding processes, aggregating features along two spatial directions. The network can quickly focus on the region of interest, and the performance of the pedestrian detection network can be effectively improved [31].

## 3. Proposed Method

**Figure 2:**
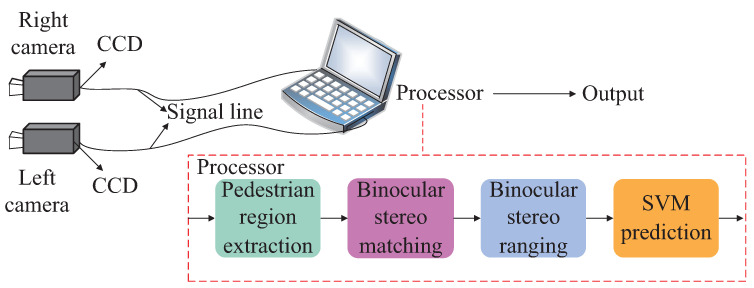
Bionic model diagram for real and pseudo pedestrian detection based on human stereo vision.

A real and pseudo pedestrian detection bionic model based on human stereo vision is designed in this paper, as shown in Figure 2. In the bionic model, the human eyes are imitated by a binocular stereo camera, which captures external visual information. The photoreceptor cells on the retina are imitated by the charge coupled device (CCD) in the camera, which converts the optical signal into an electrical signal. The electrical signal is transmitted to the processor through the signal line, and the visual center of the occipital lobe is imitated by the processor. In the processor, the pedestrian region in the image is firstly extracted by the 2D pedestrian detection network, the fusing process of binocular single vision is then simulated by the binocular stereo matching, the spatial position perception is next simulated by the binocular stereo ranging, and the real and pseudo pedestrian judgment is finally simulated by the SVM prediction.

**Figure 3:**
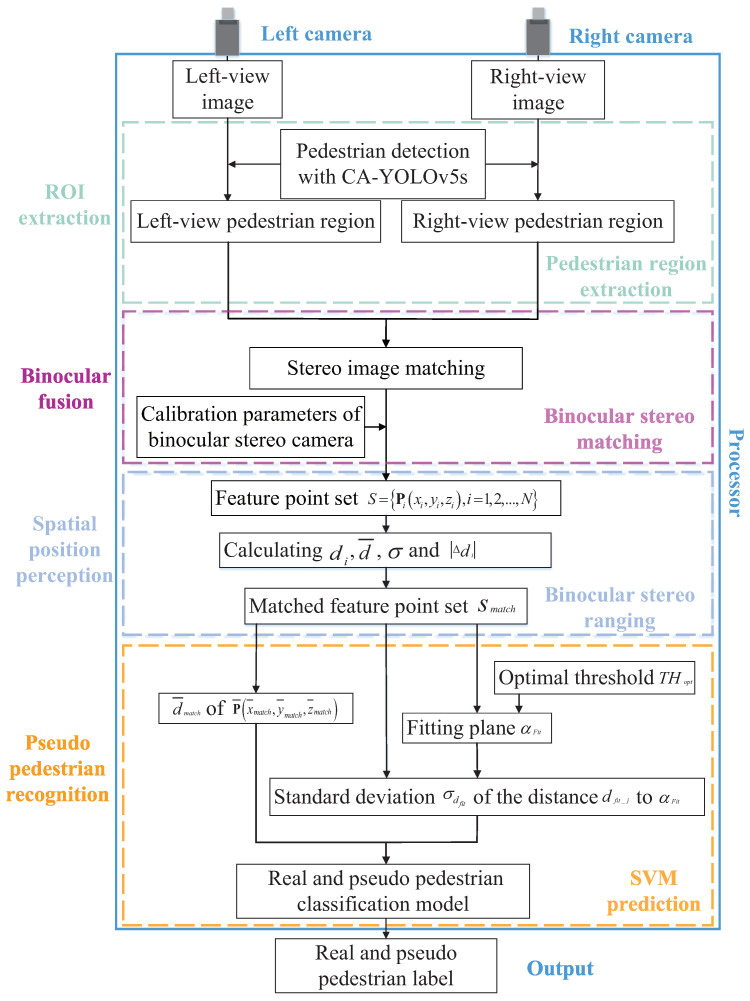
Block diagram of the real and pseudo pedestrian detection method with CA-YOLOv5s based on stereo image fusion.

To realize the function of the processor in the designed bionic model, a real and pseudo pedestrian detection method with CA-YOLOv5s based on stereo image fusion is proposed in this paper. As shown in Figure 3, the proposed method consists of four modules, pedestrian region extraction, binocular stereo matching, binocular stereo ranging and SVM prediction, which correspond to the four processes of the visual center, that is, receptive field division, binocular single vision, spatial position perception and real and pseudo pedestrian judgment. In the pedestrian region extraction module, the dual-view images containing pedestrian are collected by the binocular stereo camera, and the left-view pedestrian regions $ROI_{L}$ and the right-view pedestrian regions $ROI_{R}$ are extracted by the improved CA-YOLOv5s pedestrian detection algorithm, respectively. In the binocular stereo matching module, SURF matching [36] is performed on the $ROI_{L}$ and $ROI_{R}$ to obtain matched feature point pairs $\left(p_{Li},p_{Ri}\right),i=1,2,\ldots ,N$, and the calibration parameters $f_{L}$, $f_{R}$, R and T of the binocular stereo camera are calculated by Zhengyou Zhang’s calibration method [37]. In the binocular stereo ranging module, the feature point set $S=\left\{P_{i}\left(x_{i},y_{i},z_{i}\right),i=1,2,\ldots ,N\right\}$ corresponding to all the matched feature point pairs $\left(p_{Li},p_{Ri}\right),i=1,2,\ldots ,N$ in $ROI_{L}$ and $ROI_{R}$ is calculated according to the calibration parameters of the binocular stereo camera. The space distance $d_{i}$ between each spatial feature point $P_{i}$ and the origin of the world coordinate system, namely the optical center of the left-view camera, is calculated, the mean value $\bar{d}$ and standard deviation σ of all $d_{i}$ are derived, and the absolute difference $\left|\Delta d_{i}\right|$ between each $d_{i}$ and $\bar{d}$ is computed. The mismatched feature points are eliminated according to the relationship between $\left|\Delta d_{i}\right|$ and σ, and the matched feature point set $S_{\mathrm{match}\;}=\left\{P_{j}\left(x_{j},y_{j},z_{j}\right),j=1,2,\ldots ,M\right\}$ is obtained, M≤N. In the SVM prediction module, the mean values in *x*, *y* and *z* directions of all the points in $S_{\mathrm{match}}$ are calculated to form a new point $\bar{P}\left({\bar{x}}_{\mathrm{match}\;},{\bar{y}}_{\mathrm{match}\;},{\bar{z}}_{\mathrm{match}}\right)$, and the space distance ${\bar{d}}_{\mathrm{match}}$ is calculated to represent the space distance between the pedestrian and the camera. According to the optimal threshold $TH_{opt}$, fitting is performed on all *M* points in $S_{\mathrm{match}}$ to obtain a fitting plane $\alpha_{Fit}$. The standard deviation $\sigma_{d_{fit}}$ of the distance $d_{fit\_j}$ from each point in $S_{\mathrm{match}}$ to the fitting plane $\alpha_{Fit}$ is calculated. The ${\bar{d}}_{\mathrm{match}}$ and $\sigma_{d_{fit}}$ are input into the pre-trained real and pseudo pedestrian classification model, and real and pseudo pedestrian detection can be achieved. The proposed method solves the problem that the existing pedestrian detection algorithms cannot identify the pseudo pedestrian well, effectively reduces the number of false positive samples, and improves the accuracy of pedestrian detection.

### 3.1. Pedestrian Region Extraction

Modern deep learning-based pedestrian detection algorithm can be divided into two-stage pedestrian detection algorithm and single-stage pedestrian detection algorithm [38]. The most representative two-stage pedestrian detection algorithm is R-CNN series [39], including Fast R-CNN [40], Faster R-CNN [7], Cascade R-CNN [41], etc., with high scalability and good detection performance, but complex structure and low speed. The most representative single-stage pedestrian detection algorithm includes YOLO series [42], SSD [8], RFB [43], M2Det [44], RetinaNet [45], etc., with fast detection speed, but relatively low detection performance. However, as technical progresses in YOLO series, single-stage detection algorithms have outperformed two-stage detection algorithms not only in detection speed but also in detection accuracy. Among these single-stage detection algorithms, the YOLOv5 detection algorithm is particularly suitable for pedestrian detection because of its fast detection speed, high detection accuracy, and good deployment on hardware device [46]. There are four common detection algorithms in the YOLOv5 series, i.e., YOLOv5s, YOLOv5m, YOLOv5l and YOLOv5x. From YOLOv5s to YOLOv5x, the detection accuracy increases steadily, while the detection speed decreases rapidly and the network complexity increases significantly [47].

**Figure 4:**
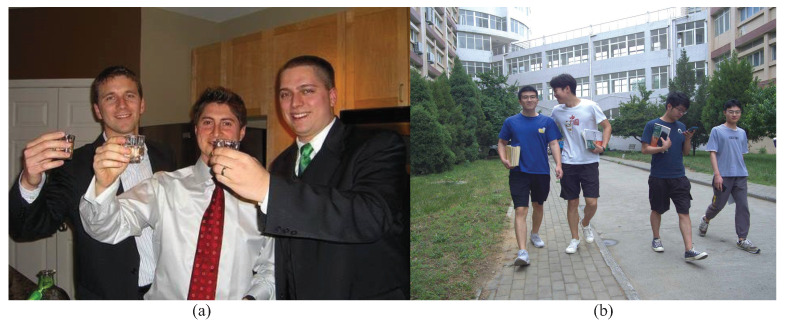
Image examples of the dataset. (**a**) Image selected from VOC dataset. (**b**) Collected image.

Eight typical object detection algorithms are selected for pedestrian detection algorithm selection and verification, namely, SSD, RFB, RetinaNet, M2Det, YOLOv3, YOLOv4, YOLOv5s and YOLOv5m. The experimental dataset consists of 17,587 images containing people selected from the public dataset VOC and 3119 pedestrian images with a resolution of 2448×2048 collected in the laboratory, totaling 20,706 images. Image samples of the dataset are shown in Figure 4. During the experiment, the same parameters are used to train the model, and the same model performance indices, that is, average precision (AP) and frame per second (FPS), are selected to evaluate the model. The experimental results are shown in Table 1. The performance indices of YOLOv5s and YOLOv5m are significantly better than the other six algorithms. For YOLOv5s, the AP is 89.35%, the FPS is 73, and the model parameter amount is 26.88 MB, while for YOLOv5m, the AP is 90.36%, the FPS is 60, and the model parameter amount is 80.23 MB. The AP of YOLOv5s is only 1.01% lower than that of YOLOv5m, but the FPS of YOLOv5s is 21.7% higher than that of YOLOv5m and the parameter amount of YOLOv5s is 66.5% lower than that of YOLOv5m. The FPS and parameter amount of YOLOv5s are significantly better than those of YOLOv5m. Therefore, on the premise of ensuring the detection accuracy, YOLOv5s with the fastest detection speed and the smallest model parameter amount is selected as the basic network for improving the pedestrian detection performance in this paper.

The attention mechanism consistent with human perception is beneficial for the pedestrian detection network to focus on pedestrian quickly. In most pedestrian detection scenes, pedestrian objects usually have characteristics of multi-scale variation and spatial position variation due to the movement of pedestrian parallel to and perpendicular to the shooting direction. Hence, both channel attention and spatial attention should be considered. Therefore, the mixed attention mechanism is selected to optimize the YOLOv5s network.

Pedestrian feature extraction was carried out in the backbone network of YOLOv5s. Glenn Jocher et al. found that the last layer of the backbone network C3 is the best choice for replacement in the process of optimizing YOLOv5s with C3 Transformer (C3TR) [47], i.e., replacing the attention module for the C3 module in the last layer of the backbone network of YOLOv5s. The CBAM mixed attention module and CA mixed attention module are used to replace the C3 module in the last layer of the backbone network of YOLOv5s. Meanwhile, the SE and ECA channel attention modules are used to complete the comparative experiment.

The improved YOLOv5s network is denoted as CBAM-YOLOv5s, CA-YOLOv5s, SE-YOLOv5s and ECA-YOLOV5s, respectively. The model parameter amount and model compression ratio are shown in Table 2. When the input image size is 640 × 640, the model parameter amount of YOLOv5s is 26.88 MB. Compared with these data, the model parameter amount of CBAM-Yolov5s is 22.50 MB, which is compressed by 16.29%. The model parameter amount of CA-YOLOv5s is 22.47 MB, which is compressed by 16.41%. The model parameter amount of SE-YOLOv5s is 27.63 MB, which is increased by 2.79%. The model parameter amount of ECA-YOLOV5s is 22.37 MB, which is compressed by 16.78%. ECA-YOLOV5s is the best, and CA-YOLOv5s is the second. The performance indices AP, recall and FPS of CBAM-YOLOv5s, CA-YOLOv5s, SE-YOLOv5s and ECA-YOLOV5s are shown in Table 3. For YOLOv5s, AP is 89.35%, recall is 82.09% and FPS is 73. Compared with this, CA-YOLOv5s is better than YOLOv5s in the AP index, CBAM-YOLOv5s and CA-YOLOv5s are better than YOLOv5s in the recall index, CBAM-YOLOv5s, CA-YOLOv5s and ECA-YOLOv5s are better than YOLOv5s in the FPS index. Only CA-YOLOv5s is better than YOLOv5s in all three indices.

**Figure 5:**
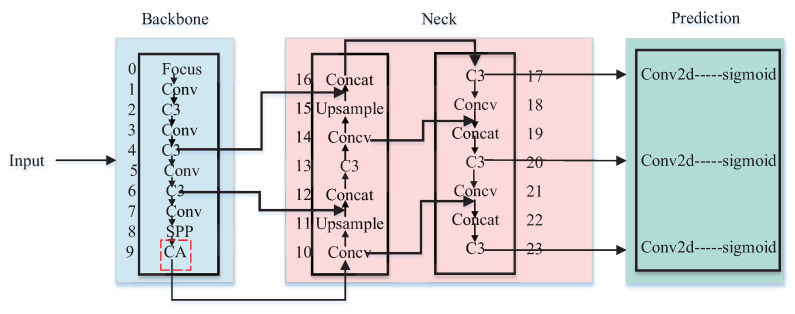
Network structure of CA-YOLOv5s.

**Figure 6:**
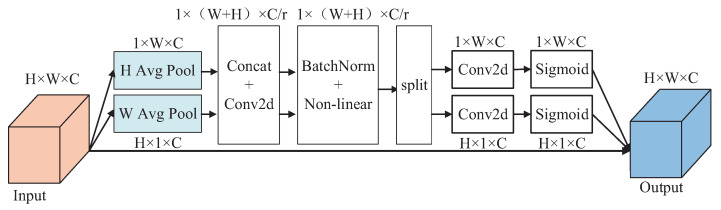
Detailed network structure of CA.

**Figure 7:**
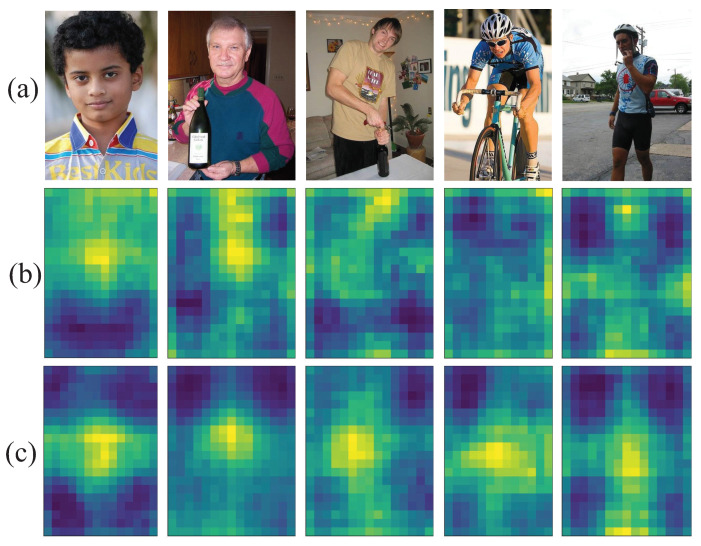
Feature visualization comparison of the last layer of the backbone network between YOLOv5s and CA-YOLOv5s. (**a**) Input image. (**b**) Feature visualization of C3 in YOLOv5s. (**c**) Feature visualization of CA in CA-YOLOv5s.

In conclusion, CA-YOLOv5s is selected as the pedestrian detection algorithm in this paper, and its network structure is shown in Figure 5, in which the C3 module in the last layer of the backbone network is replaced with the CA attention module. The detailed network structure of the CA is shown in Figure 6, in which the attention weights in height and width directions of the input feature map can be obtained respectively. The feature visualization comparison is shown in Figure 7. Compared with YOLOv5s, the features of CA-YOLOv5s are more focused on the pedestrian region.

**Figure 8:**
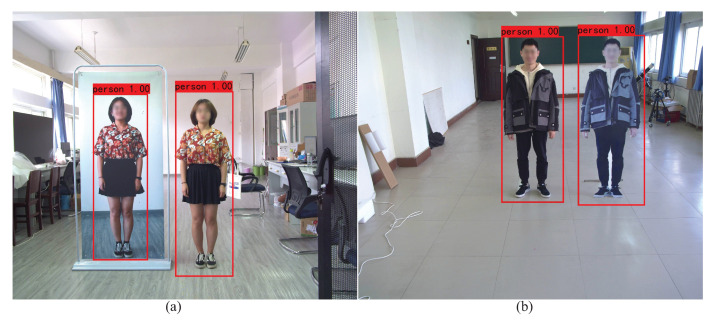
Output images of CA-YOLOv5s pedestrian detection algorithm. (**a**) Containing PPWB. (**b**) Containing PPWNB.

The output of the proposed CA-YOLOv5s pedestrian detection algorithm is shown in Figure 8. Figure 8a contains a real pedestrian and a PPWB, and Figure 8b contains a real pedestrian and a PPWNB. The output includes the bounding box of the detected pedestrian, the coordinate information of the bounding box, the label and the confidence. Table 4 illustrates the coordinates of the top left corner and the bottom right corner of the bounding box in Figure 8a, as well as the label and confidence of the detected pedestrian. As shown in Figure 8, the real pedestrian, the PPWB and the PPWNB are all detected as a pedestrian by the CA-YOLOv5s algorithm. Therefore, the real pedestrian and pseudo pedestrian should be further distinguished on this basis.

### 3.2. Binocular Stereo Matching and Ranging

**Figure 9:**
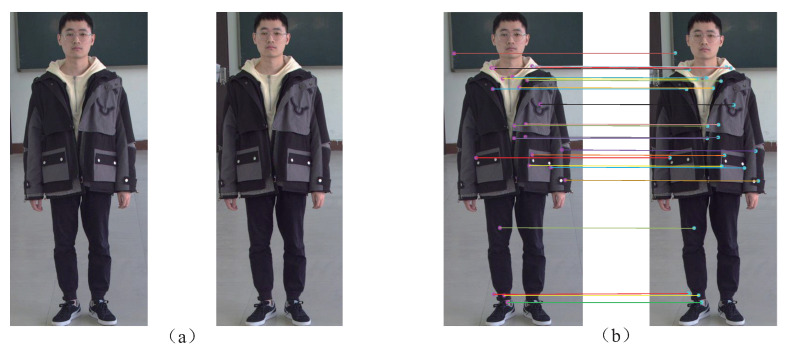
A pair of extracted pedestrian regions and their matching results. (**a**) Pedestrian region pair. (**b**) Matching results.

In the binocular stereo matching module, the extracted left-view pedestrian region $ROI_{L}$ and the right-view pedestrian region $ROI_{R}$ are stereo matched by SURF matching [36], so as to obtain the multiple matched feature point pairs $\left(p_{Li},p_{Ri}\right),i=1,2,\ldots ,N$ and the corresponding 2D coordinates $P_{Li}\left(x_{Li},y_{Li}\right)$ (in $ROI_{L}$) and $P_{Ri}\left(x_{Ri},y_{Ri}\right)$ (in $ROI_{R}$). Figure 9 shows a pair of extracted pedestrian regions and their matching result. Then the calibration parameters $f_{L}$ (left focal length), $f_{R}$ (right focal length), R (rotation matrix) and T (translation matrix) of the binocular stereo camera are calculated by Zhengyou Zhang’s calibration method [37], wherein $R=\left[\begin{array}{ccc} r_{1} & r_{2} & r_{3}\\ r_{4} & r_{5} & r_{6}\\ r_{7} & r_{8} & r_{9} \end{array}\right]$ and $T={\left[\begin{array}{ccc} t_{x} & t_{y} & t_{z} \end{array}\right]}^{T}$.

In the binocular stereo ranging module, the 3D coordinates $P_{i}\left(x_{i},y_{i},z_{i}\right)$ of the matched feature point pair $\left(P_{Li},P_{Ri}\right)$ are calculated using $P_{L}\left(x_{L},y_{L}\right)$, $P_{R}\left(x_{R},y_{R}\right)$, $f_{L}$, $f_{R}$, R and T according to Equation (17) [37]. All these spatial feature points $P_{i}\left(x_{i},y_{i},z_{i}\right)$ form a feature point set $S=\left\{P_{j}\left(x_{j},y_{j},z_{j}\right),j=1,2,\ldots ,M\right\}$. The space distance $d_{i}$ between each spatial feature point $P_{i}$ in *S* and the optical center $O_{L}$ of the left-view camera, namely the origin of the world coordinate system, is calculated according to Equation (18). The mean value $\bar{d}$ and standard deviation σ of all $d_{i}$ are derived according to Equations (19) and (20). The absolute difference $\left|\Delta d_{i}\right|$ between each $d_{i}$ and the mean value $\bar{d}$ is computed according to Equation (21).

(17) $$\left\{\begin{array}{c} x=zx_{L}/f_{L}\\ y=zy_{L}/f_{L}\\ z=\frac{f_{L}\left(f_{R}t_{x}-x_{R}t_{z}\right)}{x_{R}\left(r_{7}x_{L}+r_{8}y_{L}+f_{L}r_{9}\right)-f_{R}\left(r_{1}x_{L}+r_{2}y_{L}+f_{L}r_{3}\right)}\\ =\frac{f_{L}\left(f_{R}t_{y}-y_{R}t_{z}\right)}{y_{R}\left(r_{7}x_{L}+r_{8}y_{L}+f_{L}r_{9}\right)-f_{R}\left(r_{4}x_{L}+r_{5}y_{L}+f_{L}r_{6}\right)} \end{array}\right.$$

(18) $$d_{i}=\sqrt{x_{i}^{2}+y_{i}^{2}+z_{i}^{2}},\;i=1,2,\ldots ,N$$

(19) $$\bar{d}=\sum_{i=1}^{N}d_{i}/N,\;i=1,2,\ldots ,N$$

(20) $$\sigma =\sqrt{\sum_{i=1}^{N}{\left(d_{i}-\bar{d}\right)}^{2}/N},\;i=1,2,\ldots ,N$$

(21) $$\left|\Delta d_{i}\right|=\left|d_{i}-\bar{d}\right|$$

Since there may exist some mismatched feature points in *S*, the direct use of these feature points in the plane fitting process will lead to a large deviation in the fitting plane, and will affect the final real and pseudo pedestrian judgment. Therefore, the mismatched feature points in *S* should be eliminated first. If $\left|\Delta d_{i}\right|>\sigma$, it is considered that $P_{i}$ is not within the constraint range of the space distance standard deviation σ in *S* and is an outlier, which should be removed. If $\left|\Delta d_{i}\right|\leq \sigma$, it is considered that $P_{i}$ is within the constraint range of the space distance standard deviation σ in *S* and is a matched point, which should be reserved. Finally, a matched feature point set $S_{\mathrm{match}\;}=\left\{P_{j}\left(x_{j},y_{j},z_{j}\right),j=1,2,\ldots ,M\right\}$ is obtained, wherein M≤N. Compared with *S*, the precision of the fitting plane and the accuracy of SVM prediction can be improved by eliminating the mismatched feature points and reserving only the correctly matched feature points. So far, the pedestrian region extraction, binocular stereo matching and binocular stereo ranging have been realized, and the 3D information required for the real and pseudo pedestrian judgment is acquired.

### 3.3. SVM Prediction

In the human visual system, real and pseudo pedestrians are distinguished according to the difference of the 3D information. In the proposed method, this process can be achieved by predicting the 3D information by SVM. The mean values of all M feature points $P_{j}$ in $S_{\mathrm{match}}$ in the x,y,z directions are firstly calculated, and a new point $\bar{P}\left({\bar{x}}_{\mathrm{match}\;},{\bar{y}}_{\mathrm{match}\;},{\bar{z}}_{\mathrm{match}}\right)$ can be obtained, as expressed in Equation (22). The space distance ${\bar{d}}_{\mathrm{match}}$ of $\bar{P}$ is derived to represent the space distance between the pedestrian and the camera, as expressed in Equation (23).

(22) $$\bar{P}\left({\bar{x}}_{\mathrm{match}},{\bar{y}}_{\mathrm{match}\;},{\bar{z}}_{\mathrm{match}\;}\right)=\left(\sum_{j=1}^{M}x_{j}/M,\sum_{j=1}^{M}y_{j}/M,\sum_{j=1}^{M}z_{j}/M\right)$$

(23) $${\bar{d}}_{\mathrm{match}\;}=\sqrt{{\bar{x}}_{\mathrm{match}\;}^{2}+{\bar{y}}_{\mathrm{match}\;}^{2}+{\bar{z}}_{\mathrm{match}\;}^{2}}$$

**Figure 10:**
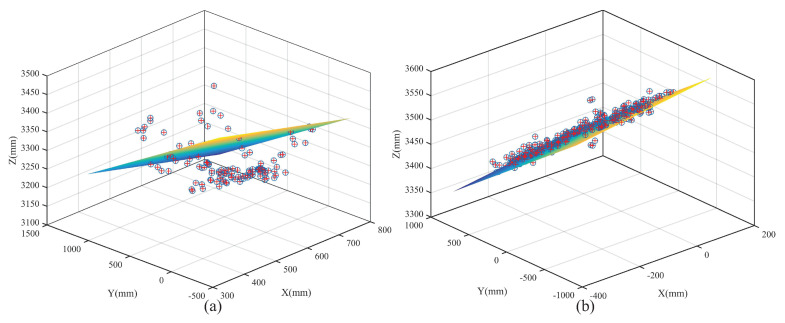
Feature point distribution of real and pseudo pedestrians. (**a**) Real pedestrian. (**b**) Pseudo pedestrian.

As shown in Figure 10, the feature points in $S_{\mathrm{match}}$ are distributed in a spatial range with a certain thickness for the real pedestrian, while the feature points in $S_{\mathrm{match}}$ are almost on the same plane for the pseudo pedestrian. Therefore, the real and pseudo pedestrian can be distinguished according to the standard deviation $\sigma_{d_{fit}}$ of the distance $d_{fit_{-}j}$ from all the feature points $P_{j}$ in $S_{\mathrm{match}}$ to their fitting plane $\alpha_{Fit}$.

For plane fitting, the random sample consensus (RANSAC) plane-fitting algorithm can fit most points to be fitted, and eliminate invalid points according to a preset threshold, which will effectively reduce the interference from matching errors [48,49,50,51]. The threshold TH should be pre-determined before the plane fitting with RANSAC. The TH can be set according to the human body error tolerance ε, which is half of the human body thickness. Not only is the human body thickness related to the chest thickness, but it is also related to the clothes to wear. In the national standard GB/T 10000 [52], a total of 47 basic human size data from six regions of the country are provided. Among them, the bare chest thickness is W∈ [0.155 m, 0.268 m], then W/2∈ [0.077 m, 0.134 m]. Considering another thickness increment, 0.03 m, of the clothes, the human body error tolerance is ε∈ [0.077 m, 0.164 m].

RANSAC plane fitting is performed on $S_{\mathrm{match}}$ according to TH, and a spatial plane $\alpha_{Fit}$ is obtained, as shown in Equation (24). The distance $d_{fit_{-}j}$ from all M feature points $P_{j}$ in $S_{\mathrm{match}}$ to the fitting plane $\alpha_{Fit}$ is computed, as shown in Equation (25). The standard deviation $\sigma_{d_{fit}}$ of $d_{fit_{-}j}$ is derived, as shown in Equation (26).

(24) Ax+By+Cz+D=0

(25) $$d_{fit\_j}=\frac{\left|Ax_{j}+By_{j}+Cz_{j}+D\right|}{\sqrt{A^{2}+B^{2}+C^{2}}}$$

(26) $$\sigma_{d_{fit}}=\sqrt{\frac{\sum_{j=1}^{M}{\left(d_{fit\_j}-\sum_{j=1}^{M}d_{fit\_j}/M\right)}^{2}}{M}}$$

**Figure 11:**
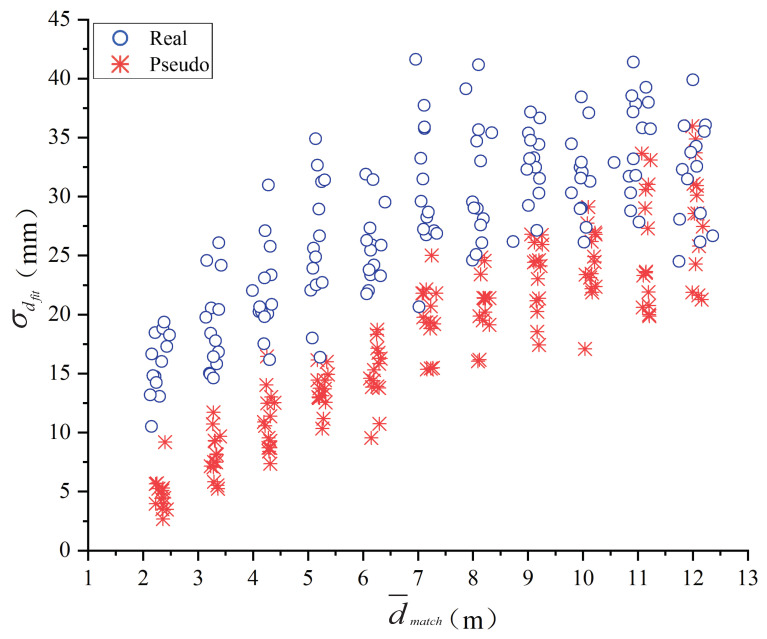
Distribution diagram of the real and pseudo pedestrian in ${\bar{d}}_{\mathrm{match}}$ and $\sigma_{d_{fit}}$ coordinates.

Figure 11 is a distribution diagram of the randomly selected real and pseudo pedestrian experimental data in the ${\bar{d}}_{\mathrm{match}}$ and $\sigma_{d_{fit}}$ coordinates. The horizontal axis ${\bar{d}}_{\mathrm{match}}$ is the space distance between the pedestrian and the camera, and the vertical axis $\sigma_{d_{fit}}$ is the standard deviation of the distance from all feature points in the human region to the fitting plane. The blue circle represents the real pedestrian, and the red asterisk represents the pseudo pedestrian. As can be seen from Figure 11, within the spatial range of 2–12 m, the experimental data conforms to the first-order linear separability law. Thus, the binary classification method can be selected for the real and pseudo pedestrian classification.

Common binary classifiers include Bayesian classifier [53], decision tree classifier [54], back propagation (BP) classifier [55] and SVM classifier [56]. As shown in Figure 11, the two input variables of the classifier are positively correlated. The input variables are required to be independent to each other for the Bayesian classifier, so it is not applicable. There are still a few points in $S_{\mathrm{match}}$ with relatively large matching error, which will lead to overfitting, so the decision tree classifier is not applicable either. Meanwhile, the binary classification problem may have multiple feasible solutions, and the BP classifier can only work out one feasible solution but not the optimal solution. The SVM classifier is the statistically optimal solution among many feasible solutions, and has higher generalization performance than the BP network. Therefore, the SVM classifier is chosen to classify the data in this paper. The training and predicting process of SVM classifier is expressed in Algorithm 1.
**Algorithm 1** The SVM training and predicting process.**Input:** Label, ${\bar{d}}_{re}$, $\sigma_{re}$ of the targets in the training set bounding box; ${\bar{d}}_{re}$, $\sigma_{re}$ of the targets in the new bounding box;**Output:** Label of the targets in the new bounding box;1:Put Label, ${\bar{d}}_{re}$, $\sigma_{re}$ of the target in the training set bounding box into the SVM for training;2:The real and pseudo classification model is obtained by SVM training;3:Send the ${\bar{d}}_{re}$ and $\sigma_{re}$ of the target in the new bounding box to the real and pseudo classification model for prediction;4:**return** The label of the target in the new bounding box.

For the training process of the SVM classifier, the input is a first-order linear separable training set $TS=\left\{\left(x_{i},y_{i}\right),i=1,2,\cdots N\right\}$, wherein, $x_{i}\left({\bar{d}}_{\mathrm{match}\_i},\sigma_{d_{fit-}i}\right)$ is the feature vector, also known as an instance; and $y_{i}\in \left\{-1,1\right\}$ is the class label of $x_{i}$. If $x_{i}$ corresponds to the real pedestrian, $y_{i}=1$; and if $x_{i}$ corresponds to the pseudo pedestrian, $y_{i}=-1$. The output is the maximal margin separation hyperplane (MMSH) and the real and pseudo pedestrian classification model.

The optimization process for linear separable SVM can be expressed by Equation (27) [57]:

(27) $$\begin{array}{c} \min_{\omega ,b}\frac{1}{2}{∥\omega ∥}^{2}\\ s.t.\;y_{i}\left(\omega \cdot x_{i}+b\right)-1\geq 0,\;i=1,2,\ldots ,N. \end{array}$$

Wherein ω and *b* are the normal vector and intercept of the separation hyperplane, and the optimal solutions $\omega^{*}$ and $b^{*}$ are the normal vector and intercept of the MMSH, which is represented by Equation (28).

(28) $$\omega^{*}\cdot x+b^{*}=0$$

The real and pseudo pedestrian classification model can be represented by Equation (29) and can be used in the predicting process of the SVM classifiers.

(29) $$f\left(x\right)=\mathrm{sgn}\left(\omega^{*}\cdot x+b^{*}\right)=\left\{\begin{array}{cc} -1, & \;\mathrm{pseudo}\;\mathrm{pedestrian}\;\\ 1, & \mathrm{real}\;\mathrm{pedestrian}\; \end{array}\right.$$

Next, the threshold TH for plane fitting is increased from 0.07 m to 0.17 m, with a step of 0.01 m. The performance indices of the SVM classification results with different TH are compared, and the optimal threshold $TH_{opt}$ is selected. In the TH optimization experiment, 64 volunteers acted as real pedestrians, and two flat panels with photos of person and two human-shaped signboards were used as pseudo pedestrians. In total, 1000 images with single pedestrian were captured, from which 783 images were randomly selected, including 394 real pedestrians and 389 pseudo pedestrians. Then, 626 images were randomly selected from the 783 images as the training set, and the remaining 157 images were used as the verification set. Table 5 shows the performance comparison of the SVM classification results for different TH.

As can be seen from Table 5, when TH = 0.15 m, the SVM classification model for real and pseudo pedestrian can achieve the best performance in both accuracy and recall, and can achieve the second-best performance in precision, which is only 0.02% lower than the best one. Therefore, the optimal threshold $TH_{opt}$ is 0.15 m. For $TH_{opt}$, the optimal solutions of the MMSH by SVM training are $\omega^{*}=\left[\begin{array}{cc} -0.69369225 & 0.26863033 \end{array}\right]$ and $b^{*}=-1.41798519$, which can be further substituted into Equations (28) and (29) to obtain the labels of the real and pseudo pedestrians in the bounding box.

## 4. Experiments

In the practical real and pseudo pedestrian detection test, two industrial cameras and a laptop are used. The Hikvision MV-CA050-11UC industrial camera has a resolution of 2448 × 2048, with a Wallis WL1608-5MP fixed-focus lens of 8 mm. The laptop is equipped with an Intel Core i7-10750H CPU, 16 GB RAM, and a Nvidia RTX2060 6G graphics card. The cell size of the calibration board is 30 mm × 30 mm. Two groups of experiments are conducted with different arrangement mode of pedestrians, i.e., equidistant arrangement mode and random arrangement mode. In the testing experiment, 71 volunteers acted as real pedestrians, and two flat panels with person photos and two human-shaped signboards were used as pseudo pedestrians. A total of 455 testing images with no occlusion were captured in the two groups of experiments, among which 212 are real pedestrians and 243 are pseudo pedestrians. In the first group of experiment with pedestrians in equidistant arrangement mode, a total of five shooting scenes were designed, that is, the pedestrian number was increased from one to five successively, and every one image was collected every one meter. In the second group of experiments with pedestrians in a random arrangement mode, a total of three shooting scenes were designed, that is, the pedestrian number was increased from three to five successively, and the pedestrians stood randomly. The main purpose of the proposed real and pseudo pedestrian detection method with CA-YOLOv5s based on stereo image fusion is to solve the problem of pseudo pedestrian detection, therefore, the testing experiments are mainly designed to verify the effect of pseudo pedestrian detection. For this reason, in the experiment, at least one pseudo pedestrian exists in each image where there is more than one pedestrian in it. As shown in Table 6, the real and pseudo pedestrian number setting is designed for five different total numbers, ranging from 1 to 5.

### 4.1. Experiments in Equidistant Arrangement Mode

**Figure 12:**
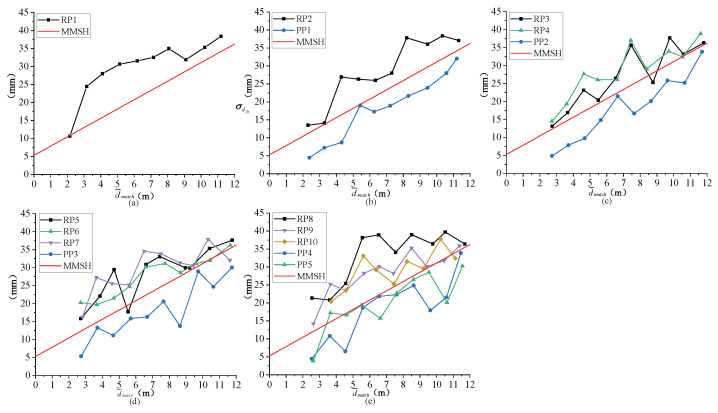
Point plots of the real and pseudo pedestrian detection results for different number of pedestrians in equidistant arrangement mode. (**a**) One pedestrian. (**b**) Two pedestrians. (**c**) Three pedestrians. (**d**) Four pedestrians. (**e**) Five pedestrians.

Figure 12 shows the point plots of the real and pseudo pedestrian detection results by the proposed method for 1–5 pedestrians arranged equidistantly, wherein the real pedestrian is represented by the label RP, and the pseudo pedestrian is represented by the label PP. A dot line represents the data points of a same pedestrian at different distances, different dot lines for different pedestrians. The MMSH is represented by a red line. If the data point is above the MMSH, it means that the pedestrian detected is a real one; if not, a pseudo one. If a RP data point is below the MMSH or a PP data point is above the MMSH, error detection occurs. For the same group of pedestrians, every image is collected every one meter at a distance from 2 m to 12 m, and 10 images can be collected for each group of pedestrians. However, when collecting images of five equidistantly arranged pedestrians, the target may not be captured due to the close distance, but the detection result will not be affected. For example, in Figure 12e, only nine images of RP10 are collected. As shown in Figure 12, the proposed method can correctly detect most data points of the real or pseudo pedestrians, but also with a small amount of error detections. The pedestrian becomes smaller with the increase in the distance, and the features become not obvious, and hence, the number of mismatched feature points increases, and the standard deviation from the feature point set to the fitting plane becomes inaccurate, resulting in the wrong classification of pedestrians. The number of error detection instances increases with the distance.

**Figure 13:**
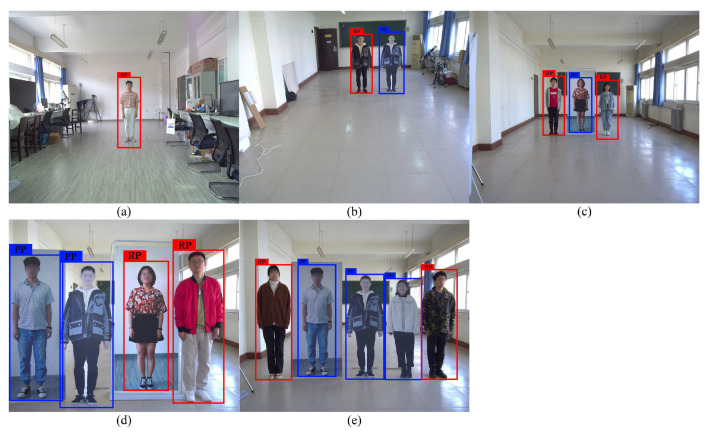
Exemplary images of the pedestrian detection outputs of the data in Table 7. (**a**) One pedestrian. (**b**) Two pedestrians. (**c**) Three pedestrians. (**d**) Four pedestrians. (**e**) Five pedestrians.

Table 7 presents the partial detailed data of Figure 13, wherein the label ‘1’ represents the real pedestrian and the label ‘−1’ represents the pseudo pedestrian. As shown in Table 7, the actual label and the predicted label are the same for most data. However, for the image with four pedestrians, the actual label for the third pedestrian is ‘−1’, while the predicted label is ‘1’, and an error detection occurs. After judging the classification of the pedestrians in the bounding box, the predicted label is combined with the coordinate information of the bounding box for output. Figure 13 shows the output images of the corresponding pedestrians in Table 7. The real pedestrian is displayed in red bounding box marked as RP, while the pseudo pedestrian is displayed in a blue bounding box marked as PP. In Figure 13d, the third (from left to right) target is PP, but detected as RP, and an error detection occurs. This small number of error detection instances is caused by matching errors.

### 4.2. Experiments in Random Arrangement Mode

**Figure 14:**
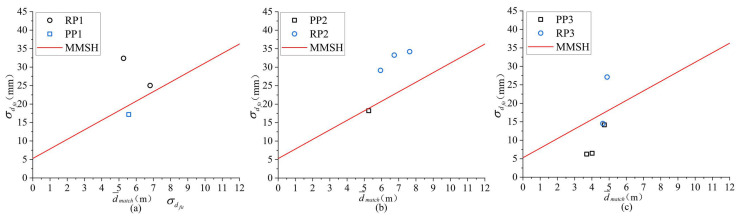
Point plots of the real and pseudo pedestrian detection results for different number of pedestrians in random arrangement mode. (**a**) Three pedestrians. (**b**) Four pedestrians. (**c**) Five pedestrians.

**Figure 15:**
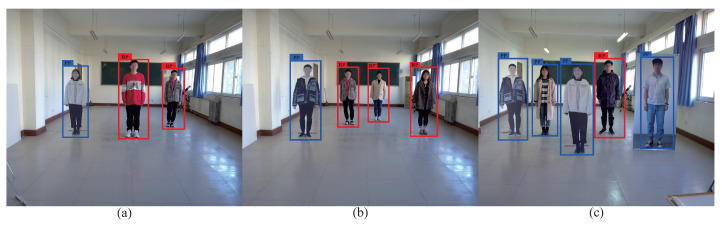
Exemplary images of the pedestrian detection output of the data in Table 8. (**a**) Three pedestrians. (**b**) Four pedestrians. (**c**) Five pedestrians.

Figure 14 shows the point plots of the real and pseudo pedestrian detection results by the proposed method for 3–5 pedestrians arranged randomly. As shown in Figure 14, the proposed method can correctly detect most data points of the real or pseudo pedestrians, but also with a small amount of error detections. Table 8 presents the detailed data of Figure 14. As shown in Table 8, the actual label and the predicted label are the same for most data. However, for the image with five pedestrians, the actual label for the second pedestrian is ‘1’, while the predicted label is ‘−1’, and an error detection occurs. Figure 15 shows the output images of the corresponding pedestrians in Table 8. In Figure 15c, the second (from left to right) target is RP, but detected as PP, and an error detection occurs. These small number of error detection instances are caused by the randomness of the feature points.

Table 9 shows the performance indices of the pedestrian detection on the 455 testing images captured in the two groups of experiments, with $TH_{opt}$ as 0.15 m. TP (True Positive) corresponds to the real label ‘1’ and the predicted label ‘1’. FN (False Negative) corresponds to the real label ‘1’ and the predicted label ‘−1’. TN (True Negative) corresponds to the real label ‘−1’ and the predicted label ‘−1’. FP (False Positive) corresponds to the real label ‘−1’ and the predicted label ‘1’. The accuracy is 93.85%, the precision is 93.81%, and the recall is 92.93%, achieving good performance for the real and pseudo pedestrian detection.

### 4.3. Contrast Experiments

The performance of the proposed method is tested and compared with seven other pedestrian detection algorithms on a same test set. Table 10 shows the performance comparison of real and pseudo pedestrian detection among different algorithms. Considering that the number of the real pedestrian is much greater than that of the pseudo pedestrians in practice, 249 images were randomly selected from the 455 testing images captured in the two groups of experiments as the testing set in the comparison experiment, of which 212 were real pedestrians and 37 were pseudo pedestrians.

As shown in Table 10, the accuracy of the seven pedestrian detection algorithms, SSD, RFB, RetinaNet, M2Det, YOLOv4, YOLOv5s and CA-YOLOv5s ranges from 85.14% to 86.35%, the precision from 85.14% to 89.69%, and the recall from 94.34% to 100%. The recalls of YOLOv4, YOLOv5s and CA-YOLOv5s are all 100%, which indicates that these three algorithms can detect all the real pedestrians in the dataset. The precisions and accuracies are all 85.14%, which means that all the pseudo pedestrians are detected as real pedestrians, i.e., the real and pseudo pedestrians cannot be distinguished. For the proposed method, the accuracy is 93.17%, the precision is 98.99%, and the recall is 92.92%. The accuracy and precision of the real and pseudo pedestrian detection are significantly superior to the other algorithms. Therefore, the real and pseudo pedestrian detection method proposed in this paper with CA-YOLOv5s based on stereo image fusion can effectively detect pseudo pedestrians, and greatly improve the accuracy and precision of the pedestrian detection network for real and pseudo pedestrian detection.

## 5. Conclusions

To solve the problem of pseudo pedestrian detection, a bionic model for the real and pseudo pedestrian detection based on human stereo vision is constructed in this paper, and a detection method with CA-YOLOv5s based on stereo image fusion for the real and pseudo pedestrian detection is proposed. In the proposed method, the YOLOv5s pedestrian detection algorithm is improved by combining with the CA attention mechanism, which not only increases the detection accuracy, but also compresses the network model size. Then, stereo matching and ranging are performed on the detected pedestrian regions based on stereo image fusion so as to obtain the 3D information of the pedestrian. Next, the trained SVM classifier is used to predict the 3D information features of the real and pseudo pedestrians extracted by the plane fitting, which can effectively distinguish between the real and pseudo pedestrians. Experimental results show that the proposed method can correctly predict the real and pseudo pedestrians and effectively solve the problem that the existing pedestrian detection algorithms cannot distinguish between real and pseudo pedestrians well.

## Figures and Tables

**Table 1:** Performance comparison of different pedestrian detection algorithms.

Algorithm	AP (%)	FPS	Parameter Amount (MB)
SSD	82.42	31	90.27
RFB	81.57	23	141.67
RetinaNet	74.63	11	138.86
M2Det	81.29	25	226.03
YOLOv3	83.89	14	234.98
YOLOv4	83.06	46	244.30
YOLOv5s	89.35	73	26.88
YOLOv5m	90.36	60	80.23

**Table 2:** Parameter amount and model compression ratio of different improved pedestrian detection algorithms with different attention modules.

No.	Detection	Model Size	Parameter Amount	Model Compression
	Algorithm		(MB)	Ratio (%)
1	YOLOv5s	640 × 640	26.88	—
2	CBAM-YOLOv5s	640 × 640	22.50	16.29
3	CA-YOLOv5s	640 × 640	22.47	16.41
4	SE-YOLOv5s	640 × 640	27.63	−2.79
5	ECA-YOLOv5s	640 × 640	22.37	16.78

**Table 3:** Performance comparison of different improved pedestrian detection algorithms with different attention modules.

No.	Detection Algorithm	AP (%)	Recall (%)	FPS
1	YOLOv5s	89.35	82.09	73
2	CBAM-YOLOv5s	89.19	82.99	74
3	CA-YOLOv5s	89.99	82.82	75
4	SE-YOLOv5s	88.34	81.34	72
5	ECA-YOLOv5s	88.94	81.32	78

**Table 4:** Output data of Figure 7a.

No.	Label and Confidence	Top Left Corner, Bottom Right Corner
1	person 1.00	(570, 585), (962, 1770)
2	person 1.00	(1155, 566), (1569, 1886)

**Table 5:** Performance comparison of SVM classification for different TH.

TH	Accuracy	Precision	Recall
0.07	78.34%	85.29%	70.73%
0.08	83.44%	91.89%	77.27%
0.09	85.99%	86.05%	88.10%
0.1	82.80%	87.50%	80.46%
0.11	84.71%	87.50%	80.77%
0.12	86.62%	87.18%	86.08%
0.13	87.26%	89.74%	85.37%
0.14	84.08%	82.28%	85.53%
0.15	91.72%	90.91%	92.11%
0.16	88.54%	90.00%	87.80%
0.17	85.35%	83.75%	87.01%

**Table 6:** Real and pseudo pedestrian number setting.

Pedestrian Number	Real Pedestrian Number	Pseudo Pedestrian Number
1	1	0
	0	1
2	1	1
	0	2
3	1	2
	2	1
	0	3
4	1	3
	2	2
	3	1
	0	4
5	1	4
	2	3
	3	2
	4	1

**Table 7:** Partial detailed data of Figure 13.

Pedestrian Number	No.	${\bar{d}}_{\mathrm{match}}$ (m)	$\sigma_{d_{\mathrm{fit}}}$ (mm)	Actual Label	Predicted Label
1	1	6.26	28.37	1	1
2	1	6.76	16.71	−1	−1
	2	6.57	31.53	1	1
3	1	6.53	26.45	1	1
	2	6.66	21.49	−1	−1
	3	6.62	26.08	1	1
4	1	3.72	11.19	−1	−1
	2	3.68	12.21	−1	−1
	3	3.71	19.68	−1	1
	4	3.67	19.81	1	1
5	1	3.75	20.98	1	1
	2	3.62	12.71	−1	−1
	3	3.70	14.54	−1	−1
	4	3.77	7.55	−1	−1
	5	3.85	26.15	1	1

**Table 8:** Detailed data of Figure 14.

Pedestrian Number	No.	${\bar{d}}_{\mathrm{match}}$ (m)	$\sigma_{d_{\mathrm{fit}}}$ (mm)	Actual Label	Predicted Label
3	1	5.57	17.17	−1	−1
	2	5.27	32.38	1	1
	3	6.81	25.01	1	1
4	1	5.26	18.21	−1	−1
	2	6.74	33.25	1	1
	3	7.64	34.21	1	1
	4	5.94	29.13	1	1
5	1	4.73	14.16	−1	−1
	2	4.64	14.51	1	−1
	3	3.70	6.24	−1	−1
	4	4.89	27.09	1	1
	5	4.02	6.47	−1	−1

**Table 9:** Detection performance on the 455 testing images.

${\mathrm{TH}}_{\mathrm{opt}}$	TP	FN	TN	FP	Accuracy	Precision	Recall
0.15	197	15	230	13	93.85%	93.81%	92.93%

**Table 10:** Performance comparison of eight different pedestrian detection algorithms on the 455 testing images.

Pedestrian Detection Algorithms	Accuracy	Precision	Recall
SSD	86.35%	87.71%	97.64%
RFB	85.14%	86.31%	98.11%
RetinaNet	85.54%	87.93%	96.23%
M2Det	85.94%	89.69%	94.34%
YOLOv4	85.14%	85.14%	100.00%
YOLOv5s	85.14%	85.14%	100.00%
CA-YOLOv5s	85.14%	85.14%	100.00%
ours	93.17%	98.99%	92.92%

## Data Availability

Not applicable.
